# Delayed diagnosis of primary hyperparathyroidism in a patient with recurrent acute pancreatitis: a case report

**DOI:** 10.3389/fmed.2026.1928811

**Published:** 2026-10-02

**Authors:** Xiaoxin Zhou, Yongji Fan, Fan Jiang

**Affiliations:** Centre of Geriatrics, Hainan Affiliated Hospital of Hainan Medical University, Hainan General Hospital, Haikou, China

**Keywords:** case report, hypercalcemia, parathyroid adenoma, primary hyperparathyroidism, recurrent acute pancreatitis

## Abstract

Primary hyperparathyroidism (PHPT) is an uncommon but clinically relevant metabolic condition associated with acute pancreatitis. We report a 32-year-old man with recurrent acute pancreatitis over approximately 4 years in whom coexisting PHPT was eventually recognized. Long-term heavy beer consumption and cholelithiasis represented important competing etiological factors. Although the exact amount and frequency of alcohol consumption could not be reliably quantified from the medical record, the patient had recently stopped drinking before the hospitalization leading to the diagnosis of PHPT. Serial abdominal ultrasonography and computed tomography did not demonstrate choledocholithiasis or biliary duct dilatation, and subsequent follow-up imaging showed no evidence of persistent biliary obstruction. Retrospective review of previous laboratory results revealed recurrent hypercalcemia and hypophosphatemia that had not prompted evaluation of parathyroid function. Subsequent testing demonstrated markedly elevated parathyroid hormone levels, and parathyroid scintigraphy and neck ultrasonography localized a right inferior parathyroid lesion. Right inferior parathyroidectomy was performed, and histopathological examination confirmed a parathyroid adenoma. On postoperative day 1, parathyroid hormone decreased markedly to 1.7 pg/mL, with serum calcium of 2.57 mmol/L. During long-term clinical follow-up, including telephone follow-up at approximately 1 and 2 years after surgery and at the most recent contact, the patient remained in good general condition without recurrent abdominal pain or acute pancreatitis. This case suggests that PHPT should be considered in patients with recurrent pancreatitis and persistent hypercalcemia, even when other potential etiological factors are present.

## 1 Introduction

Primary hyperparathyroidism (PHPT) is characterized by autonomous parathyroid hormone (PTH) secretion and is usually accompanied by hypercalcemia. Pancreatitis is an uncommon manifestation of PHPT and may be overlooked when more common etiological factors are present (1). Acute pancreatitis is most frequently associated with gallstone disease and alcohol exposure, whereas metabolic causes, including hypercalcemia, are considerably less common (2). In patients with recurrent pancreatitis, attribution to an obvious conventional risk factor may therefore delay recognition of an additional metabolic disorder. The association between PHPT and pancreatitis remains incompletely understood (1, 3). Hypercalcemia has nevertheless been proposed as a biologically plausible contributor to pancreatic injury through disturbances in calcium-dependent acinar cell signaling (4). We report a young man with recurrent acute pancreatitis in whom repeated hypercalcemia and hypophosphatemia had been documented for several years before PHPT secondary to a right inferior parathyroid adenoma was recognized. Long-term alcohol exposure and cholelithiasis complicated etiological assessment. The distinctive clinical message of this case is the delayed recognition of persistent calcium–phosphate abnormalities in a patient whose recurrent pancreatitis had initially been interpreted in the context of more common risk factors.

## 2 Case description

A 32-year-old man had a history of recurrent upper abdominal pain over approximately 4 years. His medical history included chronic hepatitis B-related cirrhosis, chronic gastritis, cholelithiasis with chronic cholecystitis, previous atrial septal defect closure, and surgery for lower-extremity varicose veins. He had consumed beer heavily for more than 10 years and had recently stopped drinking before the hospitalization leading to the diagnosis of PHPT. The medical record documented substantial alcohol intake per drinking occasion, but the exact volume and frequency of consumption and the duration of abstinence were not sufficiently recorded to permit reliable quantification. He had no history of smoking or long-term medication use. There was no known family history of hypercalcemia, nephrolithiasis, parathyroid disease, endocrine tumors, or pancreatitis.

Approximately 4 years before PHPT was diagnosed, the patient experienced his first documented episode of acute pancreatitis and required a 17-day hospitalization. Abdominal CT showed pancreatic enlargement with heterogeneous attenuation, ill-defined margins, patchy low-attenuation areas, and peripancreatic inflammatory exudation, raising concern for acute necrotizing pancreatitis (Figure 1A). No organ failure occurred, and ICU admission, mechanical ventilation, vasopressor support, or renal replacement therapy was not required. The patient improved with conservative treatment and was discharged without abdominal pain or other acute symptoms. Approximately 3 months later, follow-up CT demonstrated a pancreatic-tail pseudocyst, which subsequently regressed on later imaging. Retrospective review of laboratory results from this period also showed hypercalcemia, with serum calcium reaching 2.78 mmol/L.

**Figure 1:**
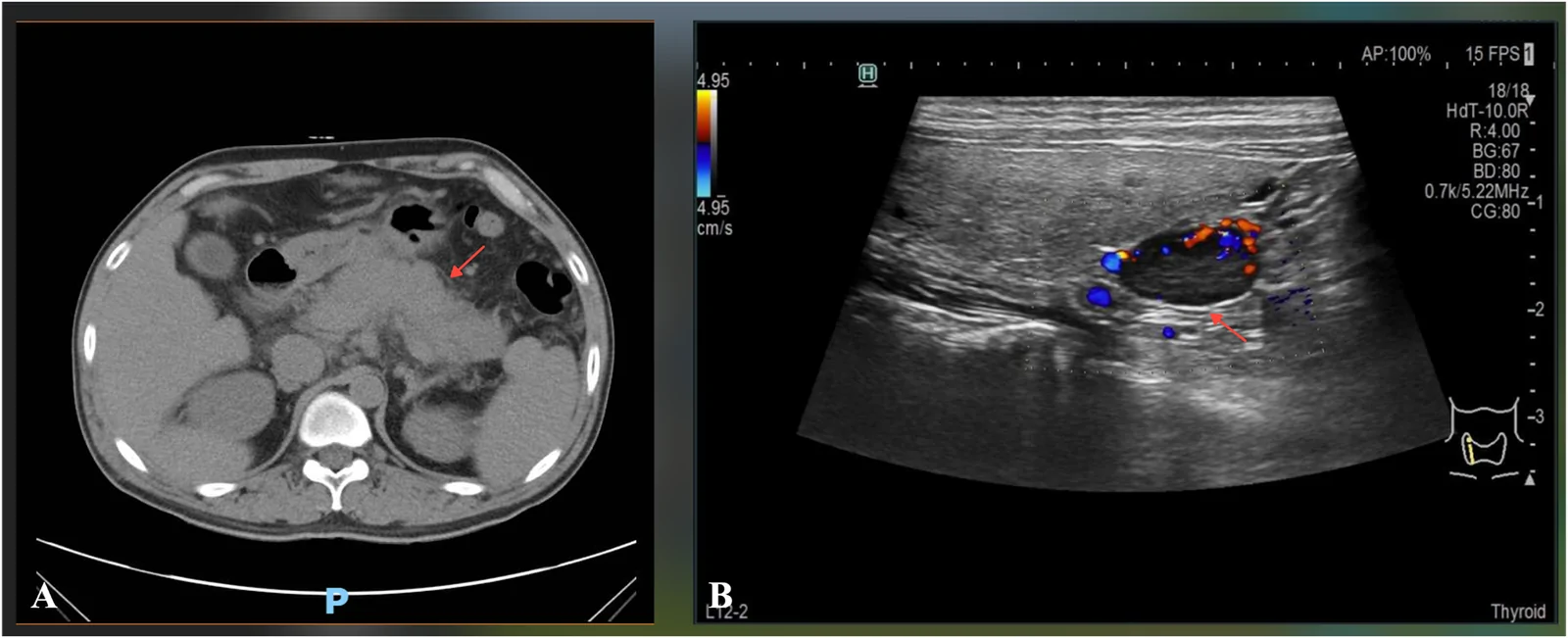
Representative imaging findings. **(A)** Abdominal contrast-enhanced CT showing pancreatic swelling, peripancreatic inflammatory exudation, and mild thickening of the anterior renal fascia. **(B)** Neck ultrasonography showing a solid hypoechoic lesion posterior to the lower pole of the right thyroid lobe, suspicious for a right inferior parathyroid lesion. Red arrows indicate the principal imaging abnormalities.

During subsequent follow-up, chronic pancreatic abnormalities persisted on imaging, with a residual low-attenuation lesion in the pancreatic tail. Intermittent abnormalities of calcium–phosphate metabolism were also documented, including a serum phosphate level as low as 0.78 mmol/L. Mild triglyceride elevation was present during this period, reaching 2.46 mmol/L.

Approximately 7 months before PHPT was diagnosed, the patient experienced another documented episode of acute pancreatitis requiring a 13-day hospitalization. Serum calcium and phosphate were 2.82 mmol/L and 0.73 mmol/L, respectively, while the triglyceride level was 1.80 mmol/L, making hypertriglyceridemia an unlikely primary explanation for this episode. Abdominal CT showed recurrent pancreatic swelling and increased peripancreatic inflammatory changes. No organ failure occurred, and ICU admission, mechanical ventilation, vasopressor support, or renal replacement therapy was not required. The patient improved with treatment and was discharged in good general condition without significant abdominal symptoms.

During this recurrence, a biliary etiology was also considered because of the history of cholelithiasis. Abdominal ultrasonography showed no dilatation of the intrahepatic or extrahepatic bile ducts and no echogenic material suggestive of choledocholithiasis. The gallbladder was normal in size without wall thickening, although a 7 × 5 mm hypoechoic lesion at the fundus was considered compatible with adenomyomatosis. Liver biochemical tests showed transient elevations in alanine aminotransferase (168.0 U/L), aspartate aminotransferase (98.7 U/L), alkaline phosphatase (198.2 U/L), *γ*-glutamyl transferase (605.3 U/L), total bilirubin (71.67 μmol/L), and direct bilirubin (58.30 μmol/L). Magnetic resonance cholangiopancreatography (MRCP) had been planned but was subsequently cancelled because of concerns related to the patient's previous atrial septal defect closure device. Endoscopic ultrasonography (EUS) and endoscopic retrograde cholangiopancreatography (ERCP) were not performed. Subsequent follow-up ultrasonography and abdominal imaging did not demonstrate gallbladder stones, choledocholithiasis, or biliary duct dilatation, and there were no clinical features of persistent biliary obstruction.

Subsequent biochemical evaluation prompted investigation for an underlying disorder of calcium metabolism. Serum PTH was markedly elevated at 301.8 pg/mL (reference range, 15.0–68.3 pg/mL), while 25-hydroxyvitamin D was 15.3 ng/mL. Further testing showed a total serum calcium level of 2.71 mmol/L with an albumin concentration of 38.4 g/L, corresponding to an albumin-corrected calcium level of approximately 2.74 mmol/L. Serum phosphate and magnesium were 0.67 and 0.61 mmol/L, respectively. Renal function was preserved, with a serum creatinine level of 60.2 μmol/L. Markedly elevated PTH was confirmed on repeat testing at 326.1 pg/mL. Twenty-four-hour urinary calcium excretion was elevated at 8.43 mmol/24 h (reference range, 2.5–7.5 mmol/24 h; urine volume, 2,800 mL). Familial hypocalciuric hypercalcemia was considered less likely because urinary calcium excretion was elevated, although a calcium-to-creatinine clearance ratio was not available. No clinical features suggestive of multiple endocrine neoplasia type 1 were documented in the available records, and genetic testing was not performed.

Parathyroid scintigraphy demonstrated focal tracer accumulation near the inferior pole of the right thyroid lobe, suggestive of a right inferior parathyroid adenoma (Figure 2A). Neck ultrasonography subsequently identified a solid lesion posterior to the lower pole of the right thyroid lobe, further supporting localization of a right inferior parathyroid lesion (Figure 1B). Taken together, the biochemical findings supported the diagnosis of PHPT, while scintigraphy and ultrasonography localized the suspected lesion to the right inferior parathyroid gland.

**Figure 2:**
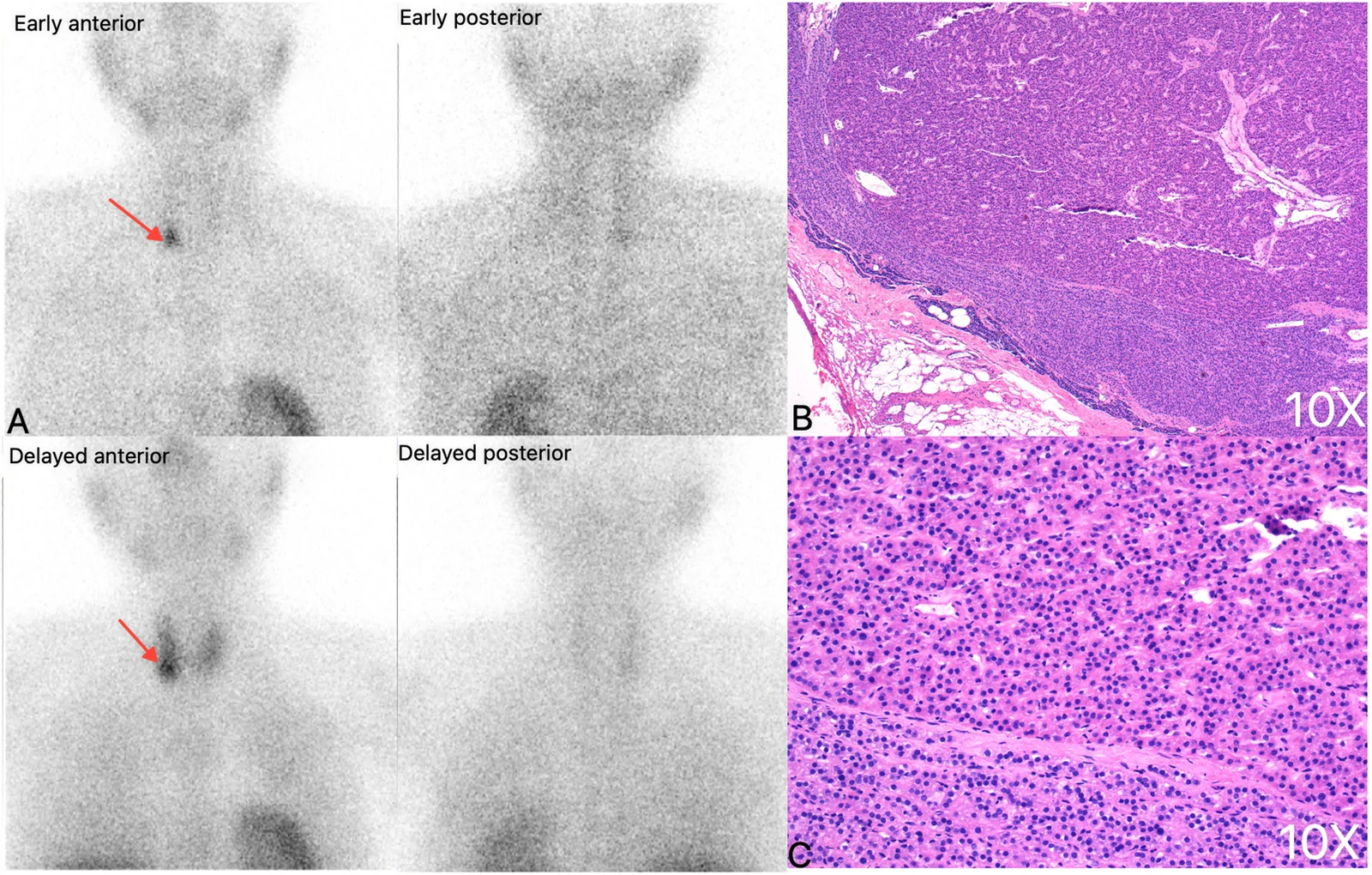
Parathyroid localization and histopathological findings. **(A)** Parathyroid scintigraphy showing early- and delayed-phase anterior and posterior images. Persistent focal tracer uptake inferior to the right thyroid lobe is indicated by red arrows. **(B)** Hematoxylin and eosin staining showing the overall architecture of the parathyroid adenoma, including the fibrous capsule and nested/trabecular growth pattern. **(C)** Hematoxylin and eosin staining showing detailed cellular morphology of the tumor. Both histopathological images were recorded as 10× magnification in the original pathology report.

During the final documented episode of acute pancreatitis before surgery, the patient presented with a 3-day history of continuous upper abdominal pain with a distending sensation. On admission, his temperature was 36.3 °C, pulse rate 127 beats/min, respiratory rate 20 breaths/min, and blood pressure 136/101 mmHg. Laboratory evaluation during this admission showed marked hyperbilirubinemia, with a total bilirubin level of 179.49 μmol/L and a direct bilirubin level of 118.01 μmol/L. The triglyceride level was 1.75 mmol/L. He was initially kept fasting and received intravenous fluid therapy and other supportive measures. Somatostatin was administered by intravenous microinfusion for approximately 2 days, followed by octreotide at 0.1 mg subcutaneously every 12 h for approximately 7 days. Esomeprazole was administered intravenously during the acute phase. Anti-infective therapy consisted initially of cefoperazone–sulbactam, followed by piperacillin–tazobactam. No persistent organ failure or new major local complication was documented, and ICU admission, mechanical ventilation, vasopressor support, or renal replacement therapy was not required.

During the same hospitalization, persistent hypercalcemia prompted calcium-lowering treatment while definitive surgery was being arranged. Salmon calcitonin was administered at 50 IU subcutaneously twice daily for approximately 10 days, together with intermittent intravenous furosemide at 20 mg. Serum calcium decreased from 3.18 mmol/L to 2.92 mmol/L, although hypercalcemia persisted.

The patient subsequently underwent right inferior parathyroidectomy under general anesthesia. Intraoperatively, the right inferior parathyroid gland was enlarged, measuring approximately 1.5 × 1.2 cm, with a well-defined margin, while the remaining parathyroid glands were not visibly enlarged. Frozen-section examination was consistent with a parathyroid adenoma. The resected specimen measured 1.3 × 0.8 × 0.7 cm and was encapsulated. Histopathological examination showed a well-circumscribed lesion surrounded by a fibrous capsule, composed predominantly of oxyphilic cells arranged in nests and trabeculae with rich vascularity, with compressed normal parathyroid tissue identified outside the capsule (Figures 2B,C). Immunohistochemical staining was positive for CK19 and chromogranin A, with a Ki-67 labeling index of approximately 1%, and negative for TTF-1. The final pathological diagnosis was a right inferior parathyroid adenoma.

The postoperative course was uneventful. No intraoperative PTH monitoring was performed. On postoperative day 1, PTH decreased markedly to 1.7 pg/mL, while serum calcium was 2.57 mmol/L and phosphate was 0.88 mmol/L. The patient did not receive routine calcium, vitamin D, or calcitriol supplementation, and no symptomatic hypocalcemia or clinically apparent hungry bone syndrome was documented. He was discharged in good general condition after a 15-day hospitalization, with stable vital signs and satisfactory wound healing.

During subsequent follow-up, the patient remained in good general condition and reported no recurrent abdominal pain or further episodes of acute pancreatitis. He continued to abstain from alcohol. No specific biliary intervention was performed, and follow-up abdominal ultrasonography and other imaging showed no gallbladder stones, choledocholithiasis, or evidence of biliary obstruction. At an earlier postoperative biochemical assessment, PTH was mildly elevated at 75.1 pg/mL without recurrent hypercalcemia. At an earlier postoperative calcium–phosphate assessment, serum calcium was 2.09 mmol/L and phosphate was 0.69 mmol/L. At approximately 1 year after surgery, serum calcium was 2.13 mmol/L, phosphate was 1.16 mmol/L, and PTH was 53.9 pg/mL. At approximately 2 years after surgery, serum calcium was 2.13 mmol/L, phosphate was 0.86 mmol/L, and PTH was 45.0 pg/mL. The patient remained in good general condition without recurrent abdominal pain or acute pancreatitis during long-term follow-up. The longitudinal changes in serum calcium, phosphate, and PTH are shown in Figure 3. The overall clinical course, diagnostic evaluation, treatment, and follow-up are summarized in Table 1.

**Figure 3:**
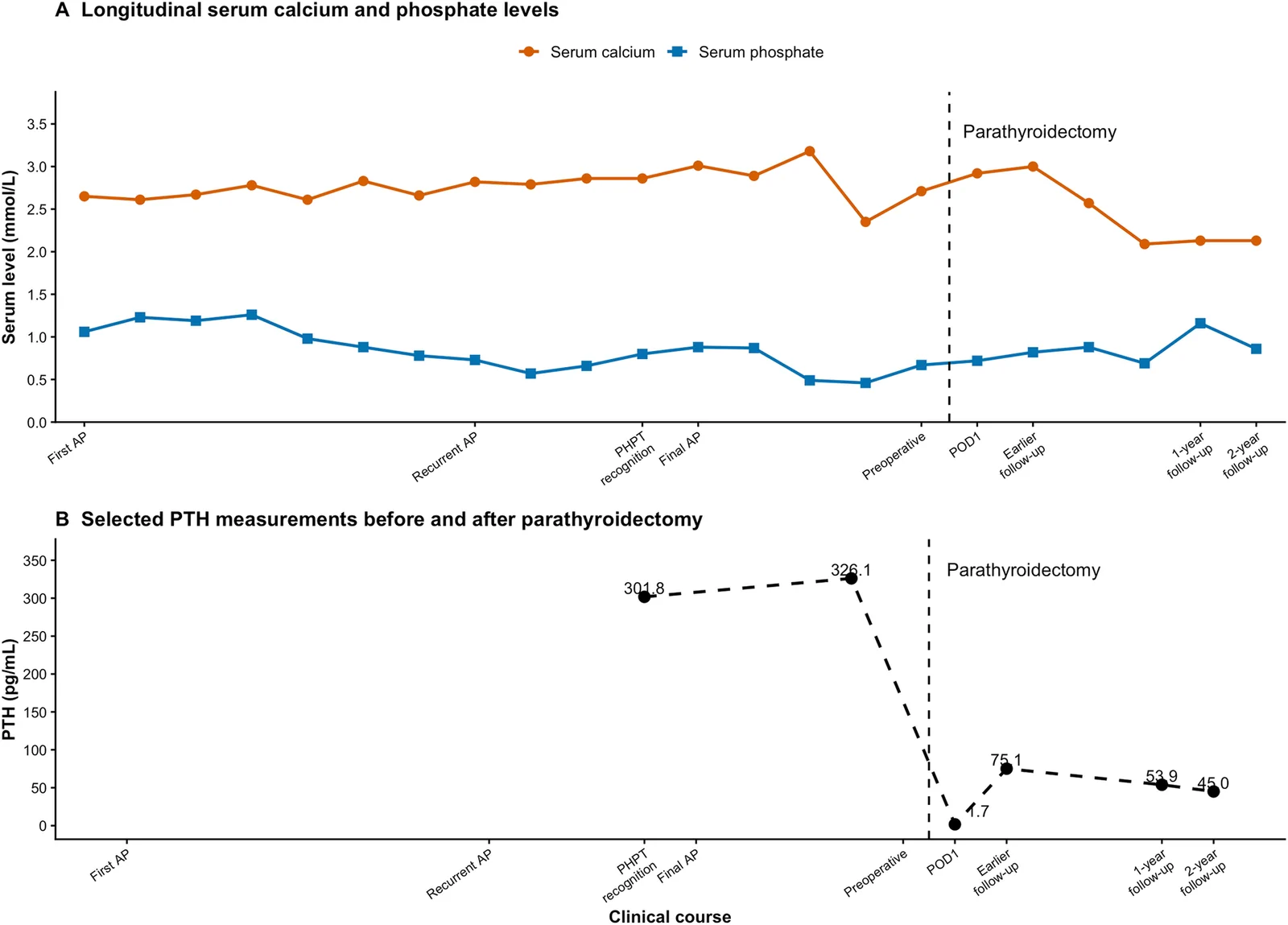
Longitudinal biochemical changes during the clinical course. **(A)** Serial serum calcium and phosphate concentrations before and after recognition of primary hyperparathyroidism and parathyroidectomy. At approximately 1 year after surgery, serum calcium and phosphate were 2.13 mmol/L and 1.16 mmol/L, respectively; at approximately 2 years after surgery, the corresponding values were 2.13 mmol/L and 0.86 mmol/L. **(B)** Selected parathyroid hormone (PTH) measurements before and after parathyroidectomy. PTH levels were 53.9 pg/mL at approximately 1 year and 45.0 pg/mL at approximately 2 years after surgery. The vertical dashed line indicates parathyroidectomy. The *x*-axis represents the clinical sequence and is not proportional to elapsed calendar time. Laboratory reference intervals varied slightly across measurement periods and are therefore not displayed as fixed reference bands.

**Table 1:** Clinical timeline of recurrent pancreatitis, diagnostic evaluation, treatment, and follow-up.

Relative time	Clinical findings	Management	Outcome/diagnosis
∼4 years before PHPT diagnosis	First documented acute pancreatitis requiring a 17-day hospitalization. CT findings raised concern for acute necrotizing pancreatitis. Hypercalcemia was retrospectively noted.	Conservative treatment.	Clinical improvement and discharge without acute abdominal symptoms. A pancreatic-tail pseudocyst was identified on follow-up imaging approximately 3 months later and subsequently regressed.
During subsequent follow-up	Persistent chronic pancreatic abnormalities on imaging. Intermittent hypercalcemia and hypophosphatemia were documented; serum phosphate decreased to 0.78 mmol/L.	Clinical follow-up.	Chronic pancreatic changes persisted; PTH was not measured.
∼7 months before PHPT diagnosis	Recurrent acute pancreatitis requiring a 13-day hospitalization. Serum calcium was 2.82 mmol/L and phosphate 0.73 mmol/L. CT showed recurrent pancreatic swelling and peripancreatic inflammation. No organ failure or ICU-level support was required.	Conservative treatment. Biliary evaluation included abdominal ultrasonography and hepatobiliary biochemical testing.	Clinical improvement and discharge in good general condition. No biliary duct dilatation or sonographic choledocholithiasis was identified. Subsequent imaging showed no evidence of persistent biliary obstruction.
PHPT diagnosis	Recurrent hypercalcemia and hypophosphatemia prompted endocrine evaluation. PTH was 301.8 pg/mL and 25-hydroxyvitamin D was 15.3 ng/mL. Twenty-four-hour urinary calcium was 8.43 mmol/24 h. Parathyroid scintigraphy and neck ultrasonography localized a suspected right inferior parathyroid lesion.	Biochemical and localization assessment.	PHPT was diagnosed; a right inferior parathyroid adenoma was suspected.
Final documented pancreatitis episode before surgery	Three-day history of continuous upper abdominal pain with a distending sensation. Temperature 36.3 °C, pulse 127 beats/min, respiratory rate 20 breaths/min, and blood pressure 136/101 mmHg. Serum calcium reached 3.18 mmol/L. Marked hyperbilirubinemia was present, with total bilirubin of 179.49 μmol/L and direct bilirubin of 118.01 μmol/L. No persistent organ failure or major local complication was documented.	Supportive treatment for acute pancreatitis. Calcitonin and intermittent furosemide were given for hypercalcemia while surgery was arranged.	Hypercalcemia decreased but persisted; no ICU admission, mechanical ventilation, vasopressor support, or renal replacement therapy was required.
Definitive treatment	Right inferior parathyroid lesion surgically resected.	Right inferior parathyroidectomy.	Histopathology confirmed parathyroid adenoma.
Postoperative day 1	PTH decreased markedly to 1.7 pg/mL; serum calcium was 2.57 mmol/L and phosphate 0.88 mmol/L.	Postoperative observation; no routine calcium, vitamin D, or calcitriol supplementation.	No symptomatic hypocalcemia or clinically apparent hungry bone syndrome was documented.
∼1 year, ∼2 years, and most recent clinical follow-up after surgery	No recurrent abdominal pain or further episodes of acute pancreatitis. At approximately 1 year, serum calcium was 2.13 mmol/L, phosphate was 1.16 mmol/L, and PTH was 53.9 pg/mL. At approximately 2 years, serum calcium was 2.13 mmol/L, phosphate was 0.86 mmol/L, and PTH was 45.0 pg/mL.	Clinical and biochemical follow-up.	The patient remained in good general condition, with no recurrent abdominal pain or acute pancreatitis reported through the most recent clinical contact.

## 3 Discussion

The main diagnostic challenge in this case was the coexistence of several plausible etiological factors for recurrent pancreatitis. Long-term heavy alcohol exposure and cholelithiasis could both have contributed to individual episodes and may have led clinicians to focus on more common causes of pancreatitis. However, retrospective review demonstrated repeated hypercalcemia and hypophosphatemia for several years before PTH was measured. The persistence of these biochemical abnormalities despite recurrent pancreatic disease suggests that an underlying disorder of calcium metabolism had remained unrecognized. Thus, the present findings support PHPT as a clinically relevant metabolic contributor, but do not establish that PHPT was the sole cause of every pancreatitis episode.

Hypercalcemia provides biological plausibility for an association between PHPT and pancreatic injury. Increased extracellular calcium may disturb intracellular calcium homeostasis in pancreatic acinar cells and promote premature trypsinogen activation, thereby increasing susceptibility to pancreatic injury (4). Dysregulated calcium signaling may activate inflammatory cascades, including NF-*κ*B and NLRP3 inflammasome pathways, thereby amplifying cytokine-mediated pancreatic inflammation (5). In this patient, hypercalcemia and hypophosphatemia were repeatedly documented during the course of pancreatic disease, and markedly elevated PTH was subsequently confirmed on repeated testing. Together with localization and pathological confirmation of a parathyroid adenoma, these findings provide clinical support for PHPT as an additional metabolic factor during the course of recurrent pancreatitis.

Competing etiologies were therefore reassessed. Triglyceride levels during the later documented acute pancreatitis episodes were 1.80 and 1.75 mmol/L, respectively, making hypertriglyceridemia an unlikely primary explanation for these attacks. Alcohol exposure remained a plausible contributor because the patient had consumed beer heavily for more than 10 years. However, he had stopped drinking before the final documented episode of acute pancreatitis, suggesting that alcohol exposure alone was insufficient to explain the recurrent attacks. The exact amount and frequency of previous alcohol consumption and the duration of abstinence could not be reliably quantified from the available records. A biliary etiology was also reconsidered because of the previous history of cholelithiasis and the marked predominantly direct hyperbilirubinemia documented during the final pancreatitis admission. Although repeated subsequent ultrasonography and other abdominal imaging showed no gallbladder stones, choledocholithiasis, biliary duct dilatation, or other evidence of persistent biliary obstruction, a transient biliary event cannot be excluded retrospectively. No specific biliary intervention was required. These findings made an ongoing biliary cause of recurrent pancreatitis less likely, although a transient passage of a gallstone during an earlier episode cannot be established retrospectively.

Parathyroidectomy resulted in a marked postoperative reduction in PTH, and no further episode of acute pancreatitis was reported during long-term clinical follow-up to the most recent contact. Previous reports have described recurrent pancreatitis preceding recognition of PHPT. Misgar et al. reported a patient in whom PHPT was diagnosed only after multiple episodes of acute pancreatitis, with no recurrent abdominal pain during follow-up after parathyroidectomy (6). Mehta et al. and Kunte et al. similarly described recurrent pancreatitis associated with parathyroid adenoma in relatively young patients (7, 8). Lenz et al. reported acute necrotizing pancreatitis as the initial manifestation of PHPT and also described pancreatic pseudocyst formation (9). Although the absence of recurrence during long-term follow-up is consistent with PHPT having been a clinically relevant contributor, the coexistence of other potential etiological factors precludes attributing the clinical course solely to parathyroidectomy. The principal clinical lesson is that persistent or recurrent hypercalcemia, particularly when accompanied by hypophosphatemia, warrants PTH measurement even when conventional risk factors for pancreatitis are already present. Earlier longitudinal review of the patient's calcium–phosphate profile might have led to earlier recognition of PHPT.

Several limitations should be acknowledged. The clinical course of the earlier pancreatitis episodes was reconstructed retrospectively from available medical records, and some diagnostic information from these episodes was incomplete. The exact alcohol exposure could not be reliably quantified. Although subsequent imaging showed no gallbladder stones, choledocholithiasis, or persistent biliary obstruction, a transient biliary event during an earlier pancreatitis episode cannot be established or excluded retrospectively. The coexistence of alcohol exposure and previous gallstone disease also limits causal attribution of the recurrent pancreatitis to PHPT alone. Although elevated 24-hour urinary calcium excretion made familial hypocalciuric hypercalcemia less likely, a calcium-to-creatinine clearance ratio was unavailable. Genetic testing for MEN1 was not performed. Long-term follow-up was primarily clinical, although biochemical measurements were available at selected postoperative time points rather than at uniform intervals. The exact common bile duct diameter was not reported in the available ultrasonography record. The precise cause of the transient hyperbilirubinemia could not be established retrospectively. Because no persistent biliary obstruction was identified on subsequent imaging, an ongoing biliary cause became less likely, although transient stone passage cannot be excluded.

## 4 Conclusion

Primary hyperparathyroidism should be considered as a potential metabolic contributor in patients with recurrent acute pancreatitis when persistent or recurrent hypercalcemia, particularly with hypophosphatemia, is present, even when conventional risk factors such as alcohol exposure or gallstone disease coexist. In the present case, acute pancreatitis recurred despite alcohol cessation, while subsequent imaging did not support an ongoing biliary cause. Following parathyroidectomy, PTH levels improved and no recurrent abdominal pain or acute pancreatitis was reported during long-term follow-up to the most recent contact. Although these findings do not establish PHPT as the sole cause of the recurrent pancreatitis, they support PHPT as an important contributing factor in this patient.

## Patient perspective

During telephone follow-up at approximately 1 and 2 years after surgery and at the most recent contact, the patient reported no recurrent abdominal pain and remained in good general condition. A formal patient-perspective statement regarding his experience of diagnosis and treatment was not obtained.

## Data Availability

The original contributions presented in the study are included in the article/Supplementary Material, further inquiries can be directed to the corresponding author.
